# Does human endometrial LGR5 gene expression suggest the existence of another hormonally regulated epithelial stem cell niche?

**DOI:** 10.1093/humrep/dey083

**Published:** 2018-04-10

**Authors:** N Tempest, A M Baker, N A Wright, D K Hapangama

**Affiliations:** 1 Liverpool Women’s Hospital NHS Foundation Trust, Liverpool L8 7SS, UK; 2 Department of Women’s and Children’s Health, Institute of Translational Medicine, University of Liverpool, Liverpool L8 7SS, UK; 3 Barts Cancer Institute, Barts and the London School of Medicine and Dentistry, Queen Mary University of London, London EC1M 6BQ, UK

**Keywords:** endometrial epithelial stem cells, leucine-rich repeat-containing G-protein-coupled receptor 5, progesterone regulation, in situ hybridisation, stem cell niche, fallopian tube

## Abstract

**STUDY QUESTION:**

Is human endometrial leucine-rich repeat-containing G-protein-coupled receptor 5 *(LGR5)* gene expression limited to the postulated epithelial stem cell niche, stratum basalis glands, and is it hormonally regulated?

**SUMMARY ANSWER:**

*LGR5* expressing cells are not limited to the postulated stem cell niche but *LGR5* expression is hormonally regulated.

**WHAT IS KNOWN ALREADY:**

The human endometrium is a highly regenerative tissue; however, endometrial epithelial stem cell markers are yet to be confirmed. LGR5 is a marker of stem cells in various epithelia.

**STUDY DESIGN, SIZE, DURATION:**

The study was conducted at a University Research Institute. Endometrial samples from 50 healthy women undergoing benign gynaecological surgery with no endometrial pathology at the Liverpool Women’s hospital were included and analysed in the following six sub-categories; proliferative, secretory phases of menstrual cycle, postmenopausal, those using oral and local progestagens and samples for *in vitro* explant culture.

**PARTICIPANTS/MATERIALS, SETTING, METHODS:**

In this study, we used the gold standard method, *in situ* hybridisation (ISH) along with qPCR and a systems biology approach to study the location of *LGR5* gene expression in full thickness human endometrium and Fallopian tubes. The progesterone regulation of endometrial *LGR5* was examined *in vivo* and in short-term cultured endometrial tissue explants *in vitro*. *LGR5* expression was correlated with epithelial proliferation (Ki67), and expression of previously reported epithelia progenitor markers (SOX9 and SSEA-1) immunohistochemistry (IHC).

**MAIN RESULTS AND THE ROLE OF CHANCE:**

*LGR5* gene expression was significantly higher in the endometrial luminal epithelium than in all other epithelial compartments in the healthy human endometrium, including the endometrial stratum basalis (*P* < 0.05). The strongest SSEA-1 and SOX9 staining was observed in the stratum basalis glands, but the general trend of SOX9 and SSEA-1 expression followed the same cyclical pattern of expression as *LGR5.* Stratum functionalis epithelial Ki67-LI and *LGR5* expression levels correlated significantly (*r* = 0.74, *P* = 0.01), however, they did not correlate in luminal and stratum basalis epithelium (*r* = 0.5 and 0.13, respectively). Endometrial *LGR5* demonstrates a dynamic spatiotemporal expression pattern, suggesting hormonal regulation. Oral and local progestogens significantly reduced endometrial *LGR5* mRNA levels compared with women not on hormonal treatment (*P* < 0.01). Our data were in agreement with *in silico* analysis of published endometrial microarrays.

**LARGE SCALE DATA:**

We did not generate our own large scale data but interrogated publically available large scale data sets.

**LIMITATIONS, REASONS FOR CAUTION:**

In the absence of reliable antibodies for human LGR5 protein and validated lineage markers for the various epithelial populations that potentially exist within the endometrium, our study does not formally characterise or examine the functional ability of the resident *LGR5*^*+*^ cells as multipotent.

**WIDER IMPLICATIONS OF THE FINDINGS:**

These data will facilitate future lineage tracing studies in the human endometrial epithelium; to identify the location of stem cells and further complement the *in vitro* functional studies, to confirm if the *LGR5* expressing epithelial cells indeed represent the epithelial stem cell population.

**STUDY FUNDING/COMPETING INTEREST(S):**

This work was supported by funding from the Wellbeing of Women project grant (RTF510) and Cancer Research UK (A14895). None of the authors have any conflicts of interest to disclose.

## Introduction

The human endometrium is a highly regenerative tissue, which undergoes over 400 cycles of menstrual shedding and re-growth in a woman’s life time. It is composed of two functionally distinct layers, the superficial stratum functionalis and the deeper stratum basalis.

The stratum functionalis is completely shed with menstruation and fully regenerated within 2 weeks, up to a thickness of 16 mm (Fleischer, 1999). This impressive regeneration implies that a stem cell population may reside within the endometrial glands. The location of stem/progenitor cells of the endometrium is postulated to be within the stratum basalis, which remains after the menstrual shedding of the stratum functionalis (Prianishnikov, 1978; Gargett *et al.*, 2016).

Of the two main endometrial specific cell types, the mesenchymal stem/progenitor cells that give rise to stromal cells are well described and studied (Gargett *et al.*, 2016). However, the evidence for an endometrial epithelial stem cell population is sparse. Previous work suggests that SSEA-1 and SOX9 expressing epithelial cell subpopulations have some ability to generate gland-like structures *in vitro* (Valentijn *et al.*, 2013; Turco *et al.*, 2017), but as yet there are no other epithelial markers with the location or functional characterisation suggestive of stem cell specificity described in the endometrium.

Leucine-rich repeat-containing G-protein-coupled receptor 5 (LGR5) is a transmembrane receptor (Barker *et al.*, 2007) which belongs to a family of glycoprotein hormone receptors (Sun *et al.*, 2009). LGR5 is a marker of stem cells in various epithelia such as the intestinal mucosa (Schuijers and Clevers, 2012), gastric mucosa (Barker *et al.*, 2010), hair follicles (Jaks *et al.*, 2008) and kidneys (Barker *et al.*, 2012). In mammary epithelium *Lgr5+* cells contribute to both luminal and basal epithelia (de Visser *et al.*, 2012) and are essential to reconstituting mammary glands from single cells (Plaks *et al.*, 2013). In the intestine, *LGR5* was shown to be a Wnt target gene, regulating epithelial regeneration with a restricted expression (visualised by *in situ* hybridisation (ISH)) in the intestinal crypt base (Barker *et al.*, 2007; Schuijers and Clevers, 2012). These basal crypt cells, were previously proposed to be an adult intestinal stem cell population, but their formal functional confirmation awaited the discovery of a specific marker (Leushacke and Barker, 2012). Subsequent work on *Lgr5+* cells from the intestine, using *in vivo* lineage tracing and a heritable-inducible lacZ reporter gene, showed that *Lgr5+* cells are long-lived, multipotent stem cells (Gerbe *et al.*, 2011), and a single *Lgr5*^*+*^ stem cell can form organoids with a gut-like architecture containing all epithelial cell types (Schuijers and Clevers, 2012).

LGR5 is expressed in the female reproductive organs. *Lgr5* marks stem/progenitor cells of the rodent ovary and the oviduct (Flesken-Nikitin *et al.*, 2013; Ng *et al.*, 2014) where it is critical for the maintenance of a functional corpus luteum and therefore, for successful pregnancy (Sun *et al.*, 2014). In immature and in ovarian hormone deprived mice, *Lgr5* is highly expressed in the single layer of epithelia lining the uterine cavity and progesterone treatment down-regulated *Lgr5*, suggesting an ovarian hormonal regulation (Sun *et al.*, 2009; Boretto *et al.*, 2017). However, mice do not menstruate, their oestrous cycle is characterised by complete reabsorption of the endometrial lining and therefore their epithelial regeneration pattern is proposed to be distinct from women (Gargett *et al.*, 2016). In the human ovary and distal Fallopian tube (fimbriae), *LGR5* expression was confirmed by quantitative reverse transcription PCR (qRT-PCR) (Ng *et al.*, 2014), with constitutive *LGR5* mRNA expression reported in healthy human endometrial epithelium throughout the menstrual cycle (Schuijers and Clevers, 2012; Krusche *et al.*, 2007).

The specificity of the available anti-human-LGR5 antibodies is disputed and in general, the antibody based protein expression data do not correlate with RNA data (Munoz *et al.*, 2012). Thus, ISH is considered as the gold standard to detect *LGR5* expressing cells in a solid tissue (Munoz *et al.*, 2012). Therefore in the human endometrium, antibody based studies need further validation (Cervello *et al.*, 2017; Gil-Sanchis *et al.*, 2013) to confirm the exact *LGR5* expressing cell population and to elucidate the function and regulation of the *LGR5* gene in those cells.

We examined the cellular localisation of *LGR5* in all epithelial compartments of the human endometrium by ISH. As the human Fallopian tube shares the same embryological origin and exists as a continuum with the endometrium, we compared the expression of *LGR5* in the epithelial mucosa of the endometrium with that of the fimbrial end of the Fallopian tube (due to its known stem cell enrichment (Auersperg, 2013)) and *Lgr5* expression (Ng *et al.*, 2014). The hormone regulation of *LGR5* in the endometrium was also studied *in vitro* and *in vivo*. Finally, published microarray datasets were interrogated to confirm *LGR5* expression and its progesterone regulation in the endometrium.

## Materials and Methods

### Human tissue

Human endometrium and tubal fimbriae was obtained from 50 women undergoing benign gynaecological surgery with no endometrial pathology at the Liverpool Women’s hospital (Supplementary Table SI), granted under Local Research Ethics (REC references; 09/H1005/55 and 11/H1005/4). Informed consent was obtained from all patients.

The cycle phase of the endometrium was assigned according to the last menstrual period and histological criteria (Noyes *et al.*, 1975; Dallenbach-Hellweg, 2012). Endometrium and the distal (fimbrial) end of the Fallopian tube samples were divided in to two pieces; one was fixed (⩾24 h in 4% (v/v) buffered formalin) and paraffin-embedded for ISH and immunohistochemical (IHC) staining, and the other immediately placed in to RNA*later* (Sigma, Dorset, UK) for RNA extraction and qRT-PCR. A further six endometrial samples from the proliferative phase of the cycle were collected in reduced serum (1%) Dulbecco’s modified Eagle’s medium (DMEM)/F12 media for short-term explant culture. ISH and IHC staining for all antibodies was analysed with specific reference to the three different endometrial epithelial compartments, the luminal epithelium (the single layer of cells that forms the luminal surface or lining of the uterine cavity), the stratum functionalis (glands in the upper two-thirds of the endometrium below the luminal epithelium, surrounded by sparse stroma) and the stratum basalis (glands in the lower one-third of the endometrium adjacent to the endo-myometrial junction, surrounded by densely packed stroma) in full-thickness endometrial tissue sections. Sequential sections were stained with pancytokeratin to confirm the assignment of epithelial compartment.

### qRT-PCR

Total RNA from tissue samples was extracted using TRIzol Plus RNA Purification System (Life Technologies, Paisley, UK), and quantified by NanoDrop ND-1000 (Thermo Fisher Scientific, Loughborough, UK). Total RNA was reverse transcribed using AMV First Strand cDNA synthesis kit (New England Bio Labs, Hertfordshire, UK) after DNase treatment (DNase I (#M0303), New England Bio Labs, Hertfordshire, UK), using the manufacturer’s protocol as previously described (Kamal *et al.*, 2016). cDNA was amplified by qPCR using iTaq SYBR Green supermix (Biorad) with the Biorad connect and the following primers: LGR5 forward (5′-CCTGCTTGACTTTGAGGAAGACC), LGR5 reverse (5′-CCAGCCATCAAGCAGGTGTTCA), GAPDH forward (5′-AATCCCATCACCATCTTCCA) and GAPDH reverse (5′-TGGACTCCACGACGTACTCA). Relative transcript expression was calculated using the ΔΔCT method, normalised to the reference gene GAPDH, using Biorad CFX manager.

### ISH

ISH for *LGR5* expression was performed as previously described (Baker *et al.*, 2015) using the RNAscope 2.5 High Definition Brown assay according to the manufacturer’s instructions (Advanced Cell Diagnostics, Hayward, CA) as detailed in supplementary methods. RNAscope probes used were *LGR5* (NM_003667.2, region 560–1589, catalogue number 311021), *POLR2A* (positive control probe, NM_000937.4, region 2514–3433, catalogue number 310451) and *dapB* (negative control probe, EF191515, region 414–862, catalogue number 310043) (Supplementary Fig. S1). *LGR5* expression was quantified according to the five-grade scoring system recommended by the manufacturer previously described (Baker *et al.*, 2015) (0 = No staining or less than 1 dot to every 10 cells (40× magnification), 1 = 1–3 dots/cell (visible at 20–40× magnification), 2 = 4–10 dots/cell, very few dot clusters (visible at 20–40× magnification), 3 = >10 dots/cell, less than 10% positive cells have dot clusters (visible at 20× magnification), 4 = >10 dots/cell, more than 10% positive cells have dot clusters (visible at 20× magnification)).

### IHC

IHC was performed on sequential tissue sections according to standard protocol as previously described (Valentijn *et al.*, 2013). Primary antibodies (mouse pan-monoclonal anti-cytokeratin (C2562, Sigma-Aldrich, Dorset, UK) at 1:4000, goat polyclonal anti-SOX9 (af3075, R&D Systems, Abingdon,UK) at 1:400, mouse monoclonal anti-Ki67 (NCL-Ki67-MM1, Novocastra, Newcastle, UK) at 1:200, mouse monoclonal anti-SSEA-1 (125601/2, Biolegend, San Diego, CA) at 1:800 dilution) were incubated overnight at 4°C in a humidified chamber. All slides were scanned using an Aperio CS2 scanner (http://www.leicabiosystems.com/digital-pathology/aperio-digital-pathology-slide-scanners/products/aperio-cs2/) and analysed using spectrum, ScanScope®.

### Analysis of IHC

Percentage of nuclear Ki67 immuno-positive cells of any intensity was evaluated as the Ki67-labelling index (Ki67-LI) (Al Kushi *et al.*, 2002; Kamal *et al.*, 2016) and the three epithelial compartments were scored separately. SOX9 and SSEA-1 immunostaining was assessed as previously described (Valentijn *et al.*, 2013), and detailed in Supplementary methods.

### Explant culture

Endometrial explant cultures were prepared from freshly collected endometrial biopsies and treated with 1 μM medroxyprogesterone acetate (MPA) or ethanol (vehicle control) for 24 h as previously described(Valentijn *et al.*, 2015). Harvested tissue after treatment was washed with PBS, immersed in RNA*later* and frozen for qRT-PCR (Valentijn *et al.*, 2015).

### Systems biology

We extended our experimental data by examining all published microarray datasets of normal, premenopausal endometrial samples from women not on hormonal treatments to explore progesterone regulation of the *LGR5* gene in the secretory compared with the proliferative menstrual cycle phase (*n* = 65) (Talbi *et al.*, 2006; Burney *et al.*, 2007; Nguyen *et al.*, 2012; Sigurgeirsson *et al.*, 2017) and in the sorted healthy normal endometrial epithelial side population cells that enrich for the endometrial epithelial stem cell population, against unsorted epithelial cells (Cervello *et al.*, 2010) (*n* = 8/group). The *in silico* methodology using oPPOSUM (http://www.cisreg.ca/oPOSSUM/), Con Tra V3, illumina’s BaseSpace Correlation Engine (BSCE; (Kupershmidt *et al.*, 2010) software; https://www.illumina.com/informatics/research/biological-data-interpretation/nextbio.html; Illumina, San Diego, CA, USA) and Ingenuity (IPA) software programmes is detailed in supplementary methods (Supplementary Table II–IV). (Mathew *et al.*, 2016; Broos *et al.*, 2011; Kupershmidt *et al.*, 2010; Cervello *et al.*, 2010; Burney *et al.*, 2007; Talbi *et al.*, 2006; Nguyen *et al.*, 2012; Sigurgeirsson *et al.*, 2017)

### Statistical methods

All statistical analyses were performed using GraphPad Prism software (Mann–Whitney *U* and one-way ANOVA was used to assess differences between groups). Spearman rank correlation was used to determine the association between pairs of variables. The criterion for significance was *P* ≤ 0.05.

## Results

### Healthy human premenopausal endometrium demonstrated dynamic spatiotemporal regulation of *LGR5* expression with high *LGR5* expressing cells in the luminal and in the stratum basalis epithelium

**Figure 1:**
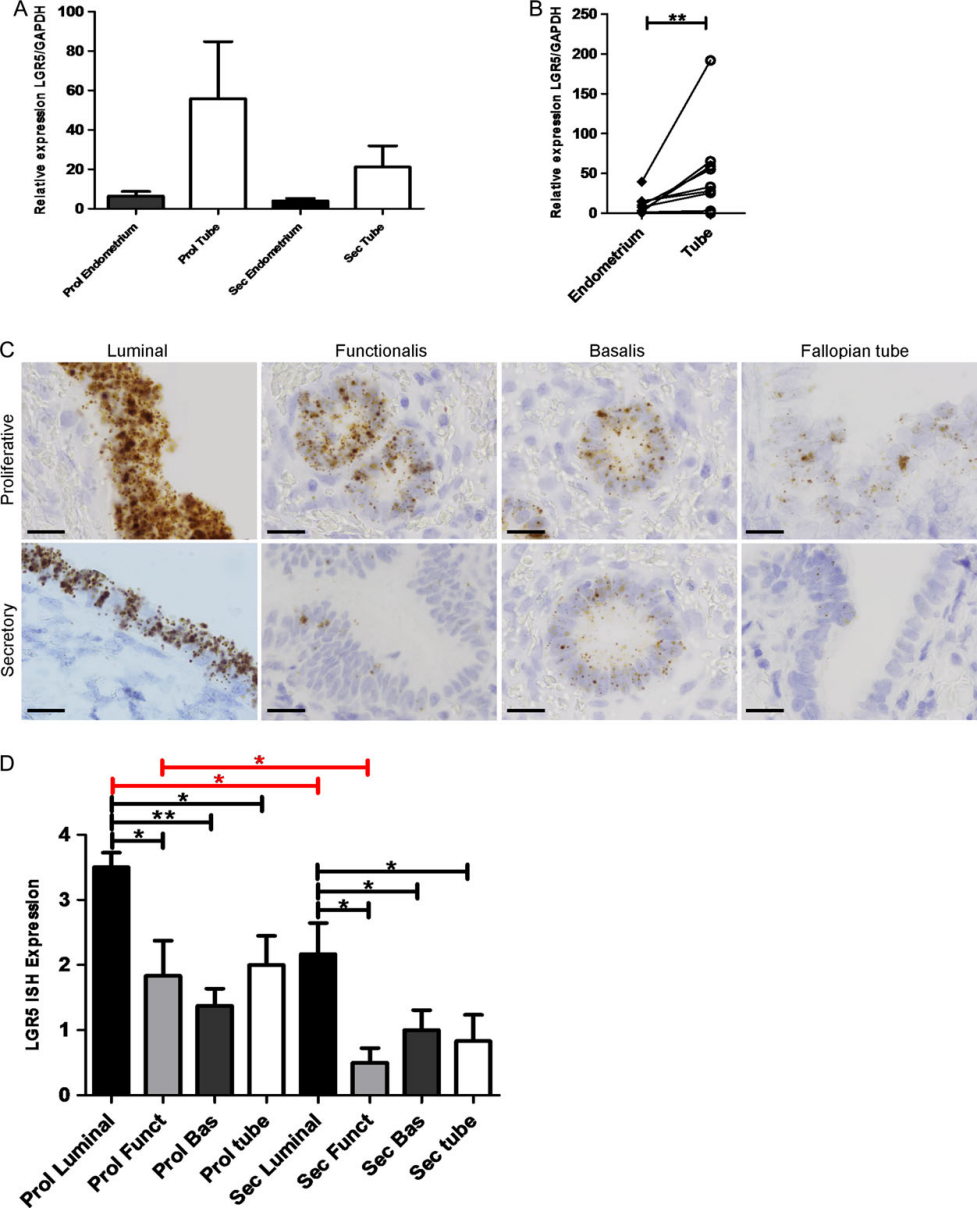
*LGR5* gene expression appears to decrease in the eutopic endometrium and Fallopian tube in the secretory phase of the menstrual cycle. (**A**) The eutopic endometrial samples and fallopian tube express apparently decreased levels of *LGR5* mRNA in the secretory phase of the menstrual cycle when compared with the proliferative phase (*n* = 21). (**B**) Fallopian tube (at any stage of the cycle) demonstrate significantly higher levels of *LGR5* mRNA than matched eutopic endometrium (*P* < 0.01) (*n* = 20). (**C**) Representative *LGR5* ISH images of Fallopian tube and luminal, stratum functionalis and stratum basalis eutopic endometrium in the proliferative and secretory stages of the menstrual cycle (All images ×1000, scale bar = 20 μm, (*n* = 15)). (**D**) Graphical representation of semi-quantitative scoring of *LGR5* ISH In the proliferative stage of the cycle, the luminal epithelium demonstrated significantly higher *LGR5* ISH staining scores than the functionalis (*P* < 0.05), basalis epithelium (*P* < 0.01) and Fallopian tube (*P* < 0.02) as well as the luminal epithelium of the secretory phase (*P* < 0.03); the proliferative functionalis had significantly higher *LGR5* scores than the secretory functionalis (*P* < 0.04); the secretory luminal epithelium showed significantly higher *LGR5* scores than the epithelia of secretory stratum functionalis (*P* < 0.03), secretory stratum basalis (*P* < 0.05) and Fallopian tube (*P* < 0.02) (*n* = 7 per group) (Prol = Proliferative, Sec = Secretory Funct = stratum Functionalis, Bas = stratum Basalis).

Full thickness whole endometrial tissue samples containing all endometrial layers and cell types from the oestrogen dominant, proliferative phase of the cycle showed a trend of higher *LGR5* mRNA expression levels (as measured by qRT-PCR) compared with the samples from the progesterone dominant secretory phase of the menstrual cycle (*P* = 0.5, Fig. 1A). *LGR5* mRNA levels were significantly higher in the stem cell rich distal Fallopian tubes than in the corresponding eutopic endometrium (*P* < 0.01, Fig. 1B). The cell type expressing *LGR5* was identified with ISH, demonstrating that *LGR5* expression was limited to the epithelial compartment in both endometrium and tube. Semi-quantitative scoring of *LGR5* expression revealed that the luminal epithelial cells expressed significantly higher levels of *LGR5* than all other epithelial compartments in the endometrium (*P* < 0.05, Fig. 1C and D) including the endometrial stratum basalis. The reduction in *LGR5* expression in the secretory phase was confirmed with ISH in the luminal (*P* = 0.03) and functionalis epithelium (*P* = 0.04) respectively (Fig. 1C and D).

### *LGR5* expression correlated with endometrial epithelial cell proliferation in the stratum functionalis epithelial compartment

**Figure 2:**
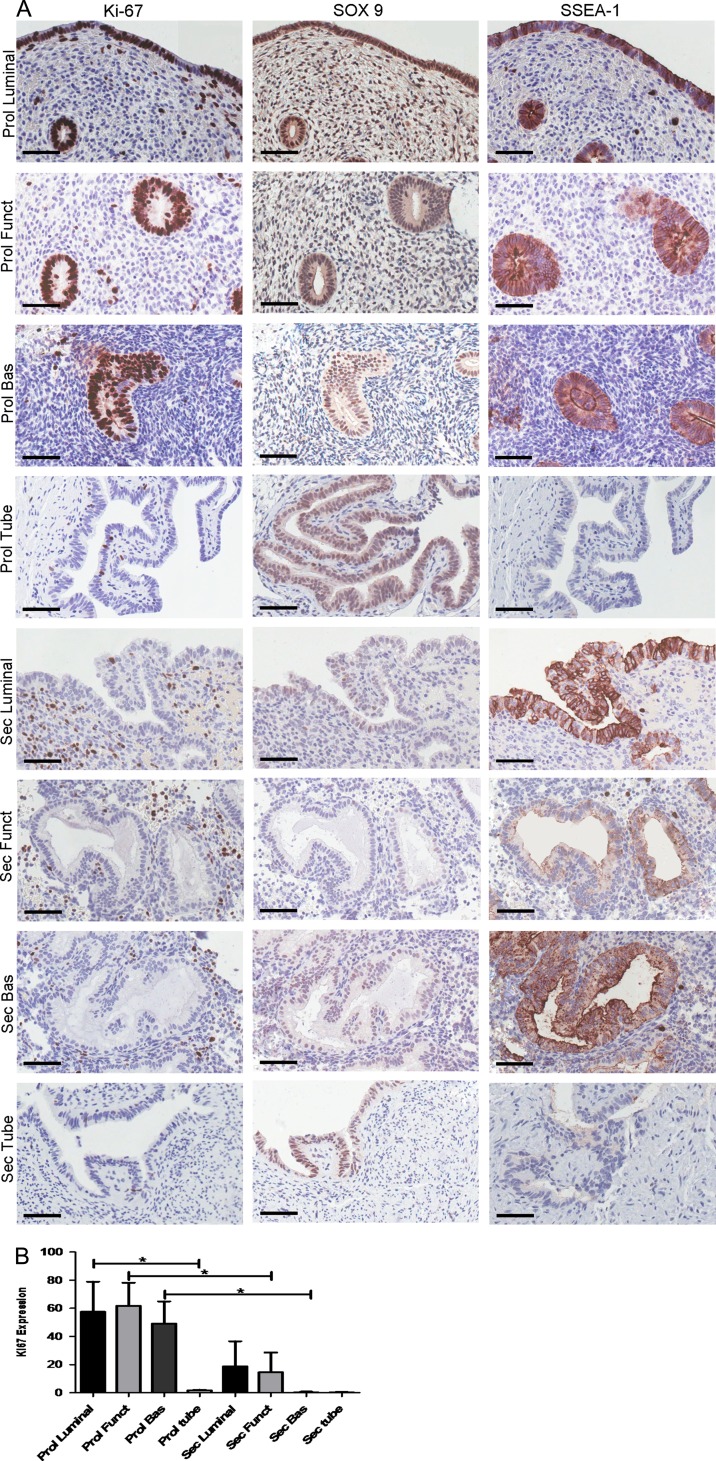
Ki67-Labelling Index (Ki67-LI) correlated with endometrial epithelial cell proliferation only in the epithelial compartment of the stratum functionalis. Epithelial *LGR5* expression scores also correlated with the expression of the previously known progenitor markers SOX9 and SSEA-1 in sequential tissue sections across the cycle. (**A**) Representative images of Ki67, SOX9 and SSEA-1 IHC in luminal, stratum functionalis, stratum basalis epithelial compartments of the eutopic endometrium and Fallopian tubes in the proliferative and secretory stages of the menstrual cycle (all images ×400, scale bar 10 μm). (**B**) Quantification of percentage Ki67-positive cells (Ki-67 LI) throughout the cycle. A minimum of 25 fields of cells were counted at ×400 magnification (*n* = 7 per group). The stratum functionalis in the proliferative phase has the highest Ki67-LI and is statistically higher than the stratum functionalis epithelium in the secretory phase (*P* < 0.05). Ki67-LI decreased dramatically in the secretory phase of the cycle in all epithelial compartments (Prol = Proliferative, Sec = Secretory Funct = stratum Functionalis, Bas = stratum Basalis).

The differences in the cellular proliferative activity in the three endometrial epithelial compartments across the menstrual cycle were demonstrated by the dynamic changes in the expression of the proliferative marker Ki67 (Fig. 2A and B) in sequential tissue sections. Epithelial Ki67-LI was highest in the proliferative phase with the maximum Ki67-LI observed in the cells of stratum functionalis glands (median 70%, range 10–100%) and the lowest Ki67-LI seen in the stratum basalis epithelium (median 30%, range 0–85%). Ki67-LI was higher in the stratum functionalis and in luminal epithelium compared with the stratum basalis glands in all phases of the cycle. In the secretory phase, Ki67-LI in all epithelial compartments decreased, with the luminal epithelial compartment demonstrating the highest Ki67-LI (median 1%, range 0–90%) and Ki67-LI was absent in the stratum basalis glands. Ki67-LI and *LGR5* expression levels only correlated significantly (*r* = 0.74, *P* = 0.01) in the stratum functionalis epithelium. The stratum basalis *LGR5* expression persisted in the secretory phase (Fig. 1D) while the corresponding Ki67-LI reactivity decreased significantly (*P* = 0.03) (Fig. 2B). The quiescent (absent Ki67-LI) atrophic postmenopausal endometrial epithelium also expressed *LGR5* (particularly the luminal epithelium) (Supplementary Fig. S2).

Epithelial proliferation (Ki67-LI) in the Fallopian tube was consistently very low throughout the cycle, contrasting with the dynamic tubal *LGR5* expression pattern (*r* = 0.23, *P* = 0.55).

### In the human endometrium, luminal and basalis epithelia share distinct patterns of co-expression of *LGR5* and the previously known progenitor markers SSEA-1 and SOX9

Sequential tissue sections were employed to examine if the cellular location of *LGR5* mRNA (by ISH) was consistent with the expression of previously described endometrial basalis progenitor markers SSEA-1 and nuclear SOX9 (by IHC, Fig. 2A). In general, SOX9 and SSEA-1 expression followed the same cyclical pattern of expression as *LGR5*: levels decreased in all three endometrial epithelial compartments and also in the tubal epithelium in the secretory phase when compared with the samples from the proliferative phase (Fig. 2A). However, out of all three endometrial epithelial compartments, the strongest *LGR5* expression was seen in the luminal epithelium (Fig. 1C and D) whereas the strongest SSEA-1 and SOX9 staining was observed in the stratum basalis glands agreeing with previous reports (Valentijn *et al.*, 2013) (Fig. 2A). It is noteworthy that the luminal staining for both SSEA-1 and SOX9 was consistently high throughout the cycle, even when their expression decreased in the stratum functionalis epithelium in the secretory phase (Fig. 2A).

In the Fallopian tube, SOX9 staining scores and *LGR5* ISH scores were high throughout the menstrual cycle, similar to the stratum basalis glands of the endometrium with only an apparent reduction in the intensity during the secretory phase (Fig. 1D). In contrast, SSEA-1 scores were very low in the tubal epithelium in all phases of the cycle. The co-expression of SSEA-1 protein and *LGR5* mRNA by ISH was further confirmed with immunofluorescence staining (Supplementary Fig. S3).

### Progestogens regulate *LGR5* expression *In vitro* and *in vivo*

**Figure 3:**
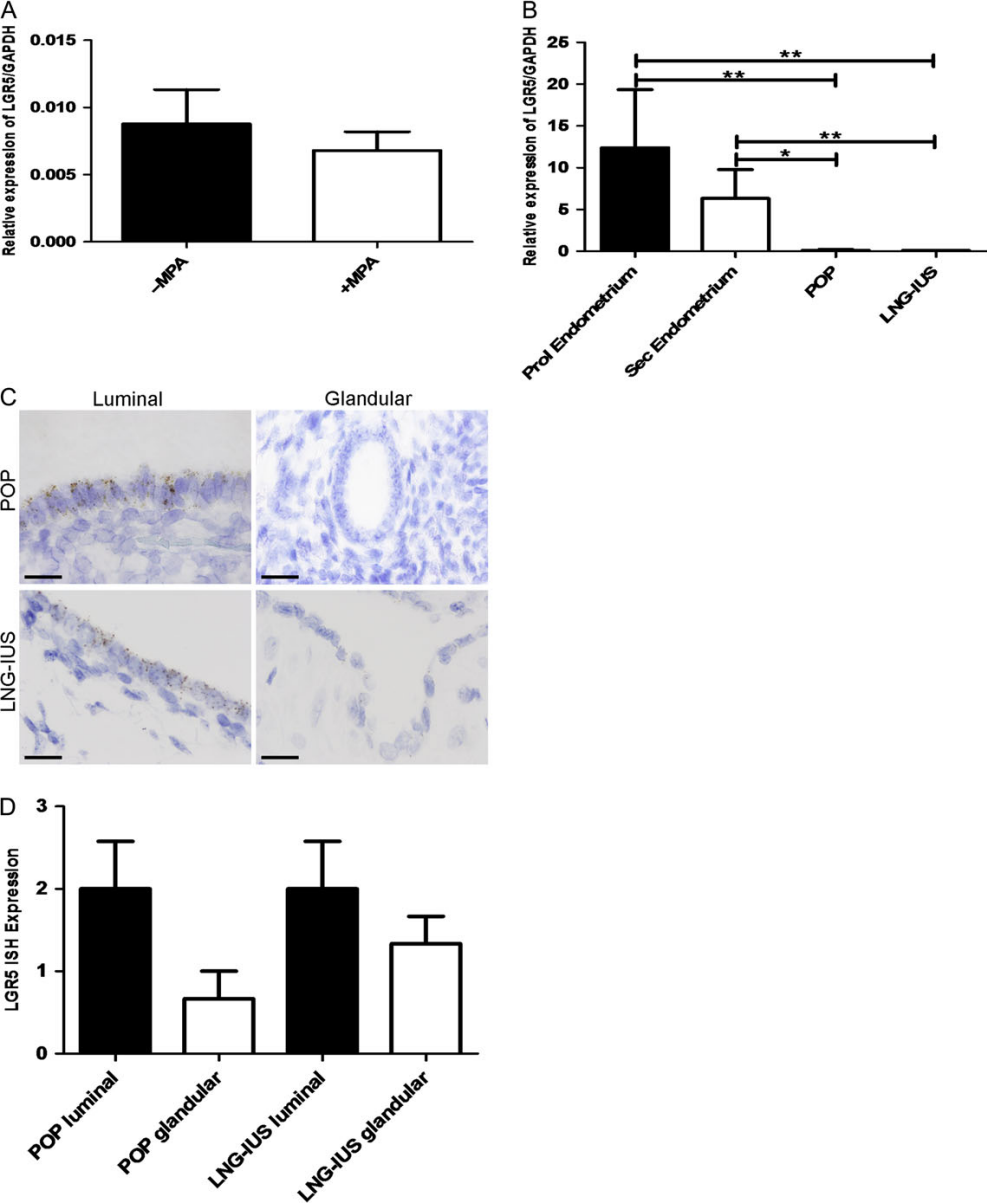
Progestagens regulate LGR5 expression *In vitro* and *In vivo*. (**A**) *LGR5* mRNA expression levels analysed by qRT-PCR. Explants treated with MPA (‘+MPA’) expressed lower *LGR5* levels relative to GAPDH when compared with the same of vehicle-treated explants (‘−MPA’) (*n* = 6 per group). (**B**) *LGR5* mRNA expression by qRT-PCR. Patients taking the oral progesterone only pill (POP) or having the levonorgestrel-releasing intrauterine system (LNG-IUS) have significantly less *LGR5* mRNA expression relative to GAPDH when compared with normal eutopic proliferative and secretory endometrium (*P* < 0.01 and *P* < 0.04, respectively). Untreated and progesterone treated (POP/LNG-IUS) (*n* = 6 per group). (**C**) Representative *LGR5* ISH images of POP treated and LNG-IUS treated luminal and glandular eutopic endometrium (All images ×1000, scale bar = 20 μm). (**D**) Graphical representation of semi-quantitative scoring of *LGR5* ISH. The luminal epithelium has more LGR5 when compared with the glandular epithelium in the POP and LNG-IUS treated samples (*n* = 6 per group).

The progestogenic regulation of *LGR5* expression was examined by treating endometrial explants *in vitro* with the synthetic progestogen MPA in short-term culture and MPA treatment decreased *LGR5* levels by 1.5-fold (Fig. 3A).

The *in vivo* effect of progestogens on the endometrial expression of *LGR5*, was tested in endometrial samples from women taking synthetic progestogen treatment (progesterone only pill, ‘POP’, or levonorgestrel-releasing intrauterine system, ‘LNG-IUS’) and a significant reduction of *LGR5* mRNA levels was observed with progestogen treatment compared with the normal eutopic endometrium of women not on hormonal treatments (*P* < 0.01, Fig. 3B). Even with these very low levels, the luminal epithelium continued to retain higher *LGR5* expression than the glands following progestogen treatment (Fig. 3C and D).

### *LGR5* expression did not correlate with epithelial cell proliferation in progesterone treated human endometrial samples

**Figure 4:**
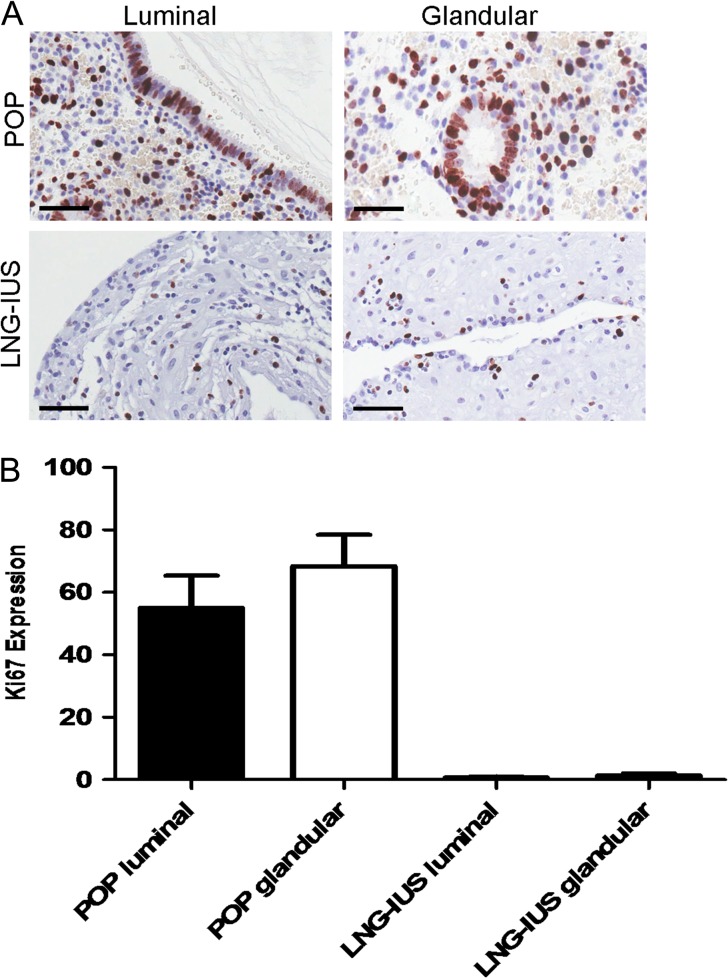
*LGR5* expression did not correlate with epithelial cell proliferation in progesterone treated human endometrial samples. (**A**) Ki67-LI IHC data, in the progesterone only pill (POP) group, the glandular and luminal epithelium showed high Ki67-LI but the levonorgestrel-releasing intrauterine system (LNG-IUS) group endometrial Ki67-LI was very low. (**B**) Representative Ki67 images of luminal and glandular eutopic endometrium from patients treated with either POP or LNG-IUS (all images ×400, scale bar = 10 μm, *n* = 6 per group).

The luminal and glandular epithelial Ki67-LI was much higher in the POP samples when compared to the LNG-IUS treated endometrium (POP luminal median 50%, range 40–75%, glands median 70%, range 50–85%, LNG-IUS luminal median 1%, range 0–1%, glands median 2% range 0–2%, Fig. 4A and B). Therefore, the Ki67-LI levels in the POP treated samples did not correlate with the levels of *LGR5* expression, whereas in the atrophic glandular and luminal epithelial cells of the LNG-IUS samples there was low levels of both *LGR5* and Ki67-LI.

### *In silico* analysis of published microarray data revealed potential *LGR5* regulating genes confirming progestagenic control

One hundred and thirty-three out of the 331 potential *LGR5* regulating transcription factors (TFs) were differentially regulated in the progesterone dominant secretory endometrium when compared with the proliferative endometrium (Supplementary Tables SII and III), supporting a role for progesterone in the regulation of *LGR5* expression. The *LGR5* gene promoter has high-affinity binding sites for the progesterone receptor, suggesting a direct regulation. The sorted human endometrial epithelial side population cells (enriched for stem cells) showed differential expression of 48 TFs that potentially regulate *LGR5* compared with the unsorted differentiated epithelial cells (Supplementary Table SIV). The analysis of the upstream regulating drugs and chemicals in IPA core analysis of the *LGR5* regulating genes that are differentially expressed in both side population epithelial cells and in the secretory phase endometrium identified progesterone, confirming our *in vitro* and *in vivo* wet-lab data (Supplementary Fig. S4) suggesting a role for progesterone in *LGR5* gene expression.

## Discussion

This is the first comprehensive study employing the gold standard method; ISH, in order to examine the cellular location of LGR5 expression in full thickness normal human endometrium. High *LGR5* expressing cells were seen in the endometrial luminal epithelium and in the stratum basalis. Healthy human endometrium shows a dynamic spatiotemporal pattern of *LGR5* expression, suggesting hormonal regulation. Endogenous and exogenous progestogens appear to inhibit *LGR5* expression in the endometrium both *in vitro* and *in vivo*, and these data and *in silico* analysis of published endometrial microarray datasets were in agreement.

Previous evidence from other epithelial tissues proposes *LGR5* expression to be limited to stem cells and thus for *LGR5* to be an epithelial stem cell marker (Kumar *et al.*, 2014). We have shown that *LGR5* was not localised to a small number of cells in the adult endometrial basalis epithelium; the proposed location of the stem cell niche (Valentijn *et al.*, 2013; Gargett *et al.*, 2016). The *LGR5* expression we have seen in the human adult endometrium, unlike in the small intestine, mimics the *Lgr5* expression pattern seen in mouse uteri (Sun *et al.*, 2009). A uniform expression of *Lgr5* was seen in the ovariectomised uterine epithelium and it is suggested that most of these remaining epithelial cells have the potential to proliferate when necessary for uterine glandular growth (Sun *et al.*, 2009). A mouse endometrial epithelial organoid system, which allowed long-term expansion of epithelium, also showed *Lgr5* gene expression (Boretto *et al.*, 2017). In contrast, in humans, the whole of the endometrial functional layer is regularly shed with menstruation, a phenomenon not relevant to most mammals including rodents. The initial step of the embryo attachment and implantation occurs at the luminal epithelium, which exists at a relatively distant location in cellular terms from the stratum basalis (up to 16 mm in the mid-secretory phase). Due to external assaults such as mechanical friction or infection, cells are continually lost and replaced from the surface of any epithelial tissue including the skin and intestine (Barker, 2014); therefore a similar daily cellular loss is likely to happen at the endometrial luminal epithelium which is exposed to the uterine cavity and external environment. The daily maintenance of this luminal epithelium may require locally positioned cells with progenitor ability. Supporting this hypothesis, rapid *Lgr5+* epithelial cell proliferation can be observed in many other organs upon tissue damage (Beumer and Clevers, 2016; Ng *et al.*, 2014).

**Figure 5:**
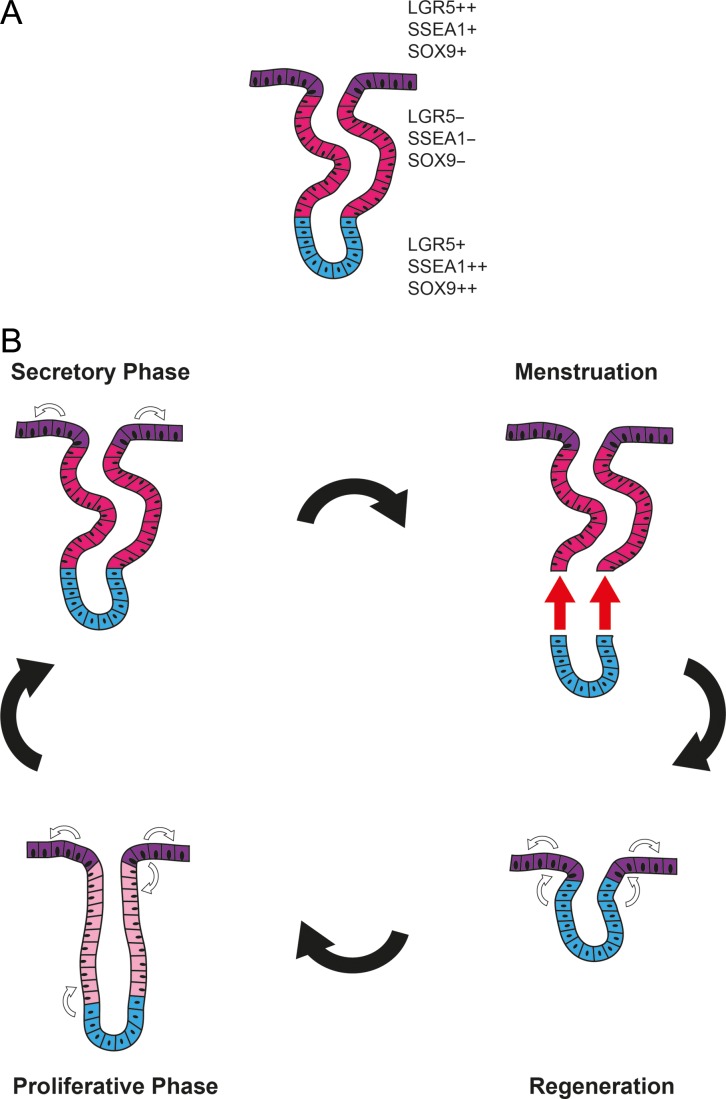
Putative model of endometrial epithelial regeneration. (**A**) The secretory phase endometrial epithelial configuration; luminal epithelia contains *LGR5*++SOX9+SSEA-1+ cells, functional glands contain *LGR5-*SOX9-SSEA-1- epithelial cells and basalis glandular cells with SOX9++SSEA-1++*LGR5*+ phenotype. (**B**) Luminal and functional layers are shed at menstruation, luminal epithelium regenerated from the stratum basalis epithelium (SOX9++SSEA-1++*LGR5*+) after menstrual shedding, subsequently, the luminal epithelium (*LGR5*++SOX9+SSEA-1+) throughout the cycle regenerates itself and possibly also contributes to the regeneration of functional glands in the proliferative phase (*LGR5*+SOX9+SSEA-1+) whilst stratum basalis glands are responsible for the regeneration of all/most of the epithelia of the stratum functionalis in the proliferative phase.

We therefore hypothesise that it is possible for the human endometrium to have more than one epithelial stem/progenitor cell pool; one residing in the basalis (SSEA-1++SOX9++*LGR5*+) supporting the massive regeneration of the functionalis after menstrual shedding or parturition; while the other (*LGR5++*SSEA-1+SOX9+) supports the embryo-implantation process, and maintains the luminal epithelial cells that are likely to be lost on a daily basis (Fig. 5). This is in agreement with the scanning electron microscopy studies of human endometrium, the endometrial injury model of the rabbit (Ferenczy and Richart, 1974) and neo-natal endometrial glandular development in humans (Cooke *et al.*, 2013). The persistent expression of the progenitor cell markers SOX9 and SSEA-1 in the luminal epithelia, with concomitant high *LGR5* expression, corroborate further with the above hypothesis (Barker and Clevers, 2010; Valentijn *et al.*, 2013). Future work examining the functional properties of endometrial epithelial cell subpopulations that are isolated from the two anatomical regions within the human endometrium, which express either high or low LGR5, SOX9 and SSEA-1, is warranted.

The two antibody based studies examining LGR5 in the human endometrium (Gil-Sanchis *et al.*, 2013; Cervello *et al.*, 2017) are in contrast to our work, in that we do not detect *LGR5* expression in the stroma but only in the pancytokeratin expressing epithelial cells and the observed proportion of epithelial cells expressing *LGR5* exceeded 1%. It should be noted that mRNA and protein levels may not necessarily correlate, and in our hands, the endometrial LGR5 protein expression using two commercially available antibodies, demonstrated non-specific staining (Supplementary Fig. S5). Therefore, agreeing with the general consensus, we concluded that the reliability of antibodies against LGR5 remains in *considerable* doubt.

Suppression of glandular regeneration and progenitor activity is postulated to occur within the progesterone-dominant, non-proliferative, secretory functional epithelium, where the lowest *LGR5* expression levels were observed. This is in agreement with a possible high *LGR5*-related stem/progenitor cell function and concurs with the *in silico* study demonstrating the differential expression of many potential regulators of *LGR5* gene in the stem cell enriched endometrial epithelial side population cells. Our interrogation of the published microarray datasets, also sought further information on the effect of progesterone on endometrial *LGR5* gene expression. We identified binding sites for progesterone, oestrogen and androgen receptors in the *LGR5* gene promoter and potential other *LGR5* gene regulators were also differentially expressed in the secretory phase endometrium. IPA core analysis re-confirmed the direct influence of progesterone on many of the identified differentially expressed *LGR5* regulators in the secretory endometrium. Considering the intricate relationship between steroid hormone receptors and their function, our experimental and *in silico* analysis data thus suggest that progesterone may directly and also indirectly regulate *LGR5* via downstream target genes. Endometrial epithelial differentiation, proliferative quiescence and inhibition of the canonical Wnt pathway in the stratum functionalis layer are known functions of progesterone (Wang *et al.*, 2009) and they were also identified as significant canonical pathways involving the differentially expressed (progesterone regulated), *LGR5* regulators in the secretory phase endometrium. This suggests a possible functional involvement of *LGR5* in the secretory endometrium that requires exploration in future studies.

In the absence of validated lineage markers for the various epithelial populations that are likely to exist within the endometrium, we cannot formally characterise the resident *LGR5*^*+*^ cells as multipotent. Lineage tracing studies need to be completed in the human endometrial epithelium to identify the location of stem cells, this will further complement the *in vitro* functional studies to confirm if *LGR5* expressing epithelial cells indeed represent the epithelial stem cell population.

## Supplementary Material

Supplementary Data

Supplementary Data

Supplementary Data

Supplementary Data

Supplementary Data

Supplementary Data

Supplementary Data

Supplementary Data

Supplementary Data

Supplementary Data
