# Supplementary material for: Does human endometrial LGR5 gene expression suggest the existence of another hormonally regulated epithelial stem cell niche?
Source: Hum Reprod. 2018 Apr 10;33(6):1052–62. doi: 10.1093/humrep/dey083 (PMC5972618; doi:10.1093/humrep/dey083)
Supplement: Supplementary Data [file dey083suppl_data.pdf]

Human Reproduction, 2018
doi:10.1093/humrep/dey083

human reproduction

SUPPLEMENTARY DATA

In situ hybridisation (ISH)

ISH for *LGR5* expression was performed as previously described (Baker *et al.*, 2015) on 5 μm sections using the RNAscope 2.5 High Definition Brown assay according to the manufacturer's instructions (Advanced Cell Diagnostics, Hayward, CA). Briefly, samples were baked at 60°C for 1 h, followed by de-paraffinisation and incubation with Pretreat 1 buffer for 10 min at room temperature (RT). Slides were boiled in Pretreat 2 buffer for 15 min, followed by incubation with Pretreat 3 buffer for 15 min at 40°C. Slides were incubated with the relevant probes for 2 h at 40°C, followed by successive incubations with Amp 1 to 6 reagents. Staining was visualised with 3,3′-diaminobenzine (DAB) 20 min, then lightly counterstained with Gill's haemotoxylin. RNAscope probes used were *LGR5* (NM_003667.2, region 560–1589, catalogue number 311021), *POLR2A* (positive control probe, NM_000937.4, region 2514–3433, catalogue number 310451) and *dapB* (negative control probe, EF191515, region 414–862, catalogue number 310043) (Supplementary Fig. S1). *LGR5* expression was quantified according to the five-grade scoring system recommended by the manufacturer and previously described (Baker *et al.*, 2015)

Analysis of immunohistochemistry (IHC)

Percentage of nuclear Ki67 immuno-positive cells of any intensity was evaluated as the Ki67-labelling index (Ki67-LI). The entire section was evaluated at ×40 magnification (minimum 25 fields) as previously described (Al Kushi *et al.*, 2002) and the three epithelial compartments were scored separately. SOX9 and SSEA-1 immunostaining was assessed by multiplying the proportion of positive cells (0 = no staining, 1 = 25%, 2 = 50%, 3 = 75% and 4 = 100%) by the staining intensity (0 = no staining, 1 = weak, 2 = moderate and 3 = strong) giving a final score between 0 and 12 (Valentijn *et al.*, 2013). All slides were scored by two independent observers who were blinded to the sample identity.

Systems Biology

A list of all (313) potential transcription factors (TFs) and regulating genes of *LGR5* were generated (Supplementary Table SII) by amalgamating three lists (Supplementary methods): (1) Over-represented TFs in the human *LGR5* gene promoter constructed with oPPOSUM (http://www.cisreg.ca/oPOSSUM/) (Mathew *et al.*, 2016). (2) All TFs binding to *LGR5* gene promotor identified with Con Tra V3 (Broos *et al.*, 2011). (3) Genes when perturbed (over expression/knockout/knockdown) affect the expression of *LGR5*, produced using Nextbio Knockout Atlas application in Illumina's BaseSpace Correlation Engine (BSCE; (Kupershmidt *et al.*, 2010) software; https://www.illumina.com/informatics/research/biological-data-interpretation/nextbio.html; Illumina, San Diego, CA, USA). The differential expression of the genes in Supplementary Table SII was examined in the secretory compared with the proliferative menstrual cycle phase in all publically available microarray datasets of normal, premenopausal endometrial samples from women not on hormonal treatments (n = 65) (Talbi *et al.*, 2006; Burney *et al.*, 2007; Nguyen *et al.*, 2012; Sigurgeirsson *et al.*, 2017) (Supplementary Table SIII) and in the only published array examining the sorted healthy normal endometrial epithelial side population cells that represent the endometrial epithelial stem cell population, against unsorted epithelial cells (Cervello *et al.*, 2010) (n = 8/group; Supplementary Table SIV) using the BaseSpace Correlation Engine. Genes common to Supplementary Table SIII and SIV were uploaded into the 'Core analysis' tool of the Ingenuity (IPA) software and upstream analysis was performed to identify progesterone target genes.

References

Al Kushi A, Lim P, Aquino-Parsons C, and Gilks CB. Markers of proliferative activity are predictors of patient outcome for low-grade endometrioid adenocarcinoma but not papillary serous carcinoma of endometrium. *Mod Pathol* 2002: **15**; 365–371.

Baker AM, Graham TA, Elia G, Wright NA, and Rodriguez-Justo M. Characterization of LGR5 stem cells in colorectal adenomas and carcinomas. *Sci Rep* 2015: **5**; 8654.

Broos S, Hulpiau P, Galle J, Hooghe B, Van Roy F, and De Bleser P. ConTra v2: a tool to identify transcription factor binding sites across species, update 2011. *Nucleic Acids Res* 2011: **39**; W74–78.

Burney RO, Talbi S, Hamilton AE, Vo KC, Nyegaard M, Nezhat CR, Lessey BA, and Giudice LC. Gene expression analysis of endometrium reveals progesterone resistance and candidate susceptibility genes in women with endometriosis. *Endocrinology* 2007: **148**; 3814–3826.

Cervello I, Gil-Sanchis C, Mas A, Delgado-Rosas F, Martinez-Conejero JA, Galan A, Martinez-Romero A, Martinez S, Navarro I, Ferro J, *et al.* Human endometrial side population cells exhibit genotypic, phenotypic and functional features of somatic stem cells. *PLoS One* 2010: **5**; e10964.

Kamal AM, Bulmer JN, DeCruze SB, Stringfellow HF, Martin-Hirsch P, and Hapangama DK. Androgen receptors are acquired by healthy postmenopausal endometrial epithelium and their subsequent loss in endometrial cancer is associated with poor survival. *Br J Cancer* 2016: **114**; 688–696.

Kupershmidt I, Su QJ, Grewal A, Sundaresh S, Halperin I, Flynn J, Shekar M, Wang H, Park J, Cui W, *et al.* Ontology-based meta-analysis of global collections of high-throughput public data. *PLoS One* 2010: **5**.

Mathew D, Drury JA, Valentijn AJ, Vasieva O, and Hapangama DK. In silico, in vitro and in vivo analysis identifies a potential role for steroid hormone regulation of FOXD3 in endometriosis-associated genes. *Hum Reprod* 2016: **31**; 345–354.

Nguyen HP, Sprung CN, and Gargett CE. Differential expression of Wnt signalling molecules between pre- and postmenopausal endometrial epithelial cells suggests a population of putative epithelial

stem/progenitor cells reside in the basalis layer. *Endocrinology* 2012: **153**; 2870–2883.

Radulescu S, Ridgway RA, Cordero J, Athineos D, Salgueiro P, Poulsom R, Neumann J, Jung A, Patel S, Woodgett J, *et al.* Acute WNT signalling activation perturbs differentiation within the adult stomach and rapidly leads to tumour formation. *Oncogene* 2013: **32**; 2048–2057.

Sigurgeirsson B, Amark H, Jemt A, Ujvari D, Westgren M, Lundeberg J, and Gidlof S. Comprehensive RNA sequencing of healthy human endometrium at two time points of the menstrual cycle. *Biol Reprod* 2017: **96**; 24–33.

Talbi S, Hamilton AE, Vo KC, Tulac S, Overgaard MT, Dosiou C, Le Shay N, Nezhat CN, Kempson R, Lessey BA, *et al.* Molecular phenotyping of human endometrium distinguishes menstrual cycle phases and underlying biological processes in normo-ovulatory women. *Endocrinology* 2006: **147**; 1097–1121.

Valentijn AJ, Palial K, Al-Lamee H, Tempest N, Drury J, Von Zglinicki T, Saretzki G, Murray P, Gargett CE, and Hapangama DK. SSEA-1 isolates human endometrial basal glandular epithelial cells: phenotypic and functional characterization and implications in the pathogenesis of endometriosis. *Hum Reprod* 2013: **28**; 2695–2708.
