# Supplementary material for: Does human endometrial LGR5 gene expression suggest the existence of another hormonally regulated epithelial stem cell niche?
Source: Hum Reprod. 2018 Apr 10;33(6):1052–62. doi: 10.1093/humrep/dey083 (PMC5972618; doi:10.1093/humrep/dey083)
Supplement: Supplementary Data [file dey083suppl_figure1.pdf]

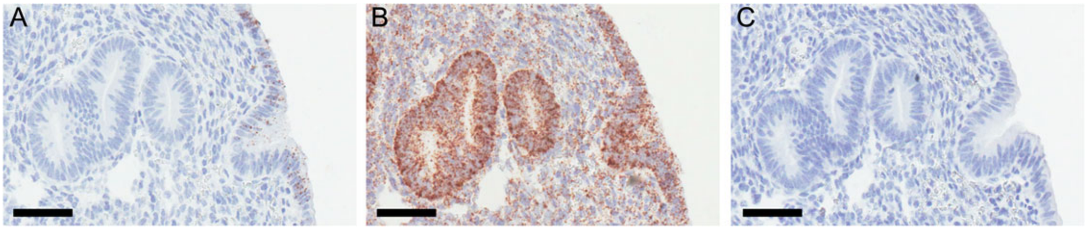

Supplementary Figure S1 Representative *LGR5* ISH showing **A**. *LGR5* Probe **B**. Positive control **C**. Negative control, images of secretory endometrium (all images ×400, scale bar = 60 μm).
