# Supplementary material for: Does human endometrial LGR5 gene expression suggest the existence of another hormonally regulated epithelial stem cell niche?
Source: Hum Reprod. 2018 Apr 10;33(6):1052–62. doi: 10.1093/humrep/dey083 (PMC5972618; doi:10.1093/humrep/dey083)
Supplement: Supplementary Data [file dey083suppl_figure2.pdf]

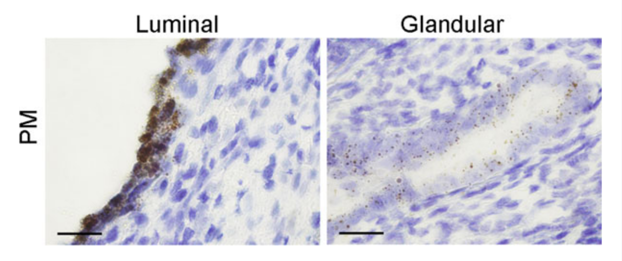

Supplementary Figure S2 Representative *LGR5* ISH images of postmenopausal (PM) luminal and glandular epithelium of healthy human endometrium (all images ×1000, scale bar = 20 μm).
