# Supplementary material for: Does human endometrial LGR5 gene expression suggest the existence of another hormonally regulated epithelial stem cell niche?
Source: Hum Reprod. 2018 Apr 10;33(6):1052–62. doi: 10.1093/humrep/dey083 (PMC5972618; doi:10.1093/humrep/dey083)
Supplement: Supplementary Data [file dey083suppl_figure3.pdf]

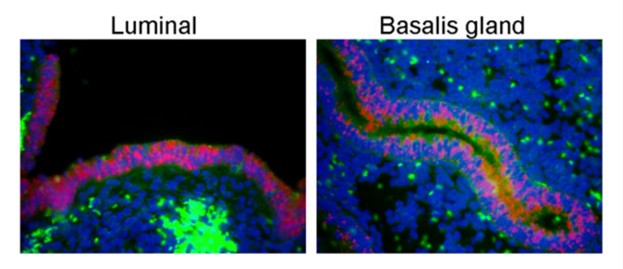


**Supplementary Figure S3** Representative co-localisation *LGR5* ISH probe (red)/anti-human SSEA-1antibody immunofluorescence (green) images of luminal and basalis epithelium of healthy human full thickness endometrium (All images ×400).
