# Supplementary material for: Does human endometrial LGR5 gene expression suggest the existence of another hormonally regulated epithelial stem cell niche?
Source: Hum Reprod. 2018 Apr 10;33(6):1052–62. doi: 10.1093/humrep/dey083 (PMC5972618; doi:10.1093/humrep/dey083)
Supplement: Supplementary Data [file dey083suppl_figure4.pdf]

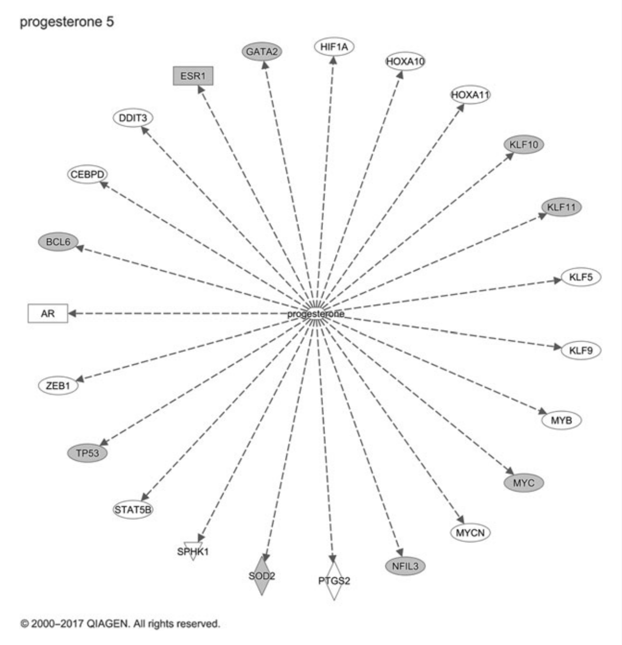

Supplementary Figure S4 Progesterone target genes that are likely to be regulating LGR5 in the secretory endometrium and in the endometrial epithelial side population cells. The potential LGR5 regulating genes that are differentially expressed in the epithelial side population cells compared with the unsorted epithelial cells (Supplementary Table SIII) and in the secretory endometrium compared with the proliferative phase endometrium (Supplementary Table SIV) were included in the IPA upstream analysis to identify those which are downstream targets of progesterone.
