# Supplementary material for: Does human endometrial LGR5 gene expression suggest the existence of another hormonally regulated epithelial stem cell niche?
Source: Hum Reprod. 2018 Apr 10;33(6):1052–62. doi: 10.1093/humrep/dey083 (PMC5972618; doi:10.1093/humrep/dey083)
Supplement: Supplementary Data [file dey083suppl_figure5.pdf]

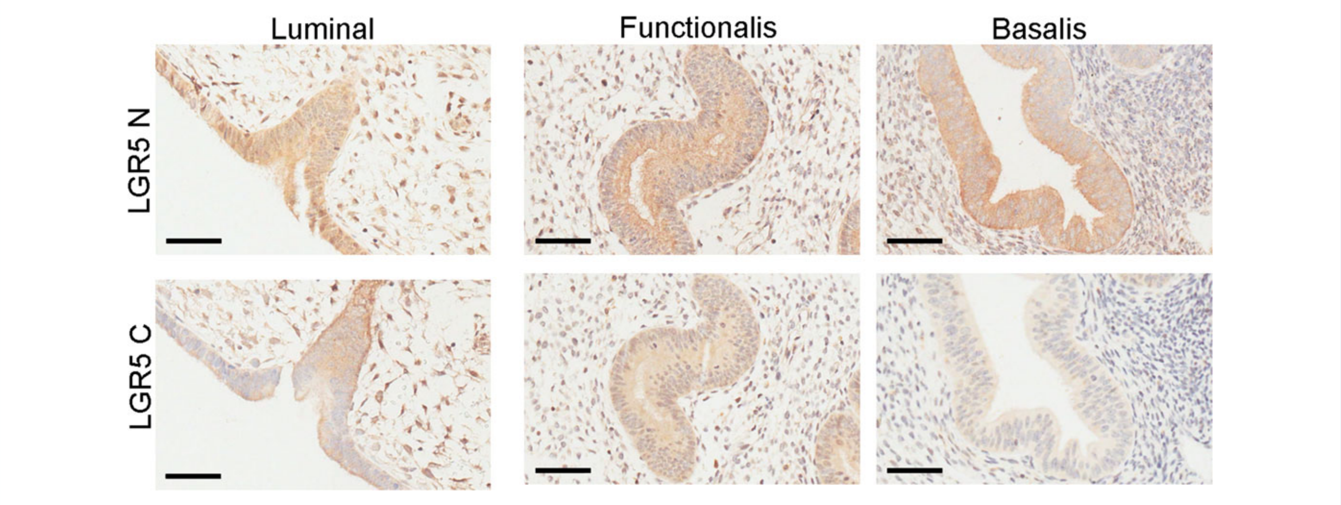

Supplementary Figure S5 Representative images of staining pattern demonstrated with two commercially available IHC LGR5 antibodies, in luminal, stratum functionalis and stratum basalis of the human endometrium; LGR5 Antibody (centre) (LGR5 C) rabbit polyclonal (AP2745f) at 1:100 dilution and LGR5 Antibody (N-term) (LGR5 N) rabbit polyclonal (AP2745a) (all images ×400, scale bar = 10 μm).
