# Supplementary material for: Does human endometrial LGR5 gene expression suggest the existence of another hormonally regulated epithelial stem cell niche?
Source: Hum Reprod. 2018 Apr 10;33(6):1052–62. doi: 10.1093/humrep/dey083 (PMC5972618; doi:10.1093/humrep/dey083)
Supplement: Supplementary Data [file dey083suppl_table1.pdf]

Supplementary Table SI **Demographics details of the patients included in the study.**

Sample no.	Stage of cycle/treatment	Age (y)	BMI (kg/m^2)	Smoker	Parity
1	Proliferative	37	22.8	Yes	2
2	Proliferative	31	43.9	No	1
3	Proliferative	57	22	No	3
4	Proliferative	42	25.6	Yes	2
5	Proliferative	39	36.9	No	4
6	Proliferative	50	21	No	4
7	Proliferative	32	27.8	Yes	2
8	Proliferative	45	36.4	No	3
9	Proliferative	43	40.5	No	3
10	Proliferative	48	37.3	No	2
11	Proliferative	37	39.2	No	2
12	Proliferative	44	29.6	No	1
13	Proliferative	44	24.5	No	2
14	Secretory	32	26.6	No	2
15	Secretory	45	31.6	No	0
16	Secretory	35	32	No	3
17	Secretory	45	26.1	No	0
18	Secretory	47	22.6	No	0
19	Secretory	44	30.7	Yes	2
20	Secretory	37	21.7	No	4
21	Secretory	41	18.9	Yes	2
22	Secretory	39	22.4	Yes	6
23	Secretory	21	25.9	No	0
24	Secretory	46	25.4	Yes	1
25	Secretory	33	26.1	No	2
26	Secretory	40	33	No	2
27	POP	36	23.9	No	4
28	POP	33	27.4	Yes	3
29	POP	25	20.9	No	0
30	POP	31	25.8	Yes	2
31	POP	37	25.7	No	0
32	POP	43	24.2	yes	2
33	LNG-IUS	46	26.2	No	3
34	LNG-IUS	45	22.5	No	2
35	LNG-IUS	35	24.4	No	1
36	LNG-IUS	33	32.7	Yes	3
37	LNG-IUS	33	25.9	No	5
38	Explant	26	33.4	Yes	2
39	Explant	44	30.7	Yes	2
40	Explant	27	33.7	No	2
41	Explant	40	33	No	2
42	Explant	43	25.4	No	2
43	Explant	47	24.2	Yes	0
44	Explant	48	28.6	No	2
45	PM	69	24.7	No	4
46	PM	69	26.8	No	3
47	PM	67	32.8	No	4

Continued

Supplementary Table SI *Continued*

Sample no.	Stage of cycle/treatment	Age (y)	BMI (kg/m^2)	Smoker	Parity
48	PM	66	24.9	No	3
49	PM	52	39.6	No	2
50	PM	74	35.6	No	3

POP, progesterone only pill; LNG-IUS, levonorgestrel-releasing intrauterine system; PM, postmenopausal.
