# Supplementary material for: Does human endometrial LGR5 gene expression suggest the existence of another hormonally regulated epithelial stem cell niche?
Source: Hum Reprod. 2018 Apr 10;33(6):1052–62. doi: 10.1093/humrep/dey083 (PMC5972618; doi:10.1093/humrep/dey083)
Supplement: Supplementary Data [file dey083suppl_table4.pdf]

Supplementary Table SIV **Potential LGR5 regulating genes that are differentially expressed in the endometrial side population cells. The only published microarray study examining the sorted healthy normal endometrial epithelial side population cells that represent the primitive endometrial epithelial stem cell population, against the unsorted, therefore more differentiated epithelial cells (Cervello *et al.*, 2010) (*n* = 8/group) was considered and included in the Nextbio-meta-analysis to identify the potential LGR5 regulating genes in the epithelial stem cells. Forty eight out of the 313 LGR5 regulating genes identified in the Supplementary table II were differentially expressed in the epithelial side population.**

Biosets versus Genes record downloaded from Correlation Engine (2017/05/19 04:26:14)
Bioset 1: Endometrial epithelium – sorted side population cells _vs_ unsorted epithelial cells (Study: Side population cells of the human endometrial epithelium and stroma)

https://liverpool.ussc.informatics.illumina.com/c/search/adv.nb?ids=241789

Gene	EntrezGene ID	Gene description	Score	*P*-value	Activity type	Activity value
CCL4	6351	chemokine (C-C motif) ligand 4	99.42137	0.0165	Fold change	14.4
NEUROG1	4762	neurogenin 1	96.90486	0.0206	Fold change	−9.09
SOX17	64321	SRY (sex determining region Y)-box 17	85.69715	0.0437	Fold change	−4.46
SOD2	6648	superoxide dismutase 2, mitochondrial	77.15811	0.0063	Fold change	3.68
BCL6	604	B-cell CLL/lymphoma 6	76.38836	0.0097	Fold change	3.63
NR3C1	2908	nuclear receptor subfamily 3, group C, member 1 (glucocorticoid receptor)	74.32844	0.0005	Fold change	3.48
BCR	613	breakpoint cluster region	72.14903	0.0392	Fold change	−3.38
TBX3	6926	T-box 3	70.3809	0.039	Fold change	3.26
GATA2	2624	GATA binding protein 2	70.23754	0.0003	Fold change	−3.25
RUNX1	861	runt-related transcription factor 1	65.49732	0.0295	Fold change	3.06
FOXA2	3170	forkhead box A2	64.45625	0.0015	Fold change	−3.02
KLF10	7071	Kruppel-like factor 10	62.58292	0.0043	Fold change	2.95
NFIL3	4783	nuclear factor, interleukin 3 regulated	62.46955	0.0166	Fold change	2.94
CDX2	1045	caudal type homeobox 2	57.99993	0.0166	Fold change	−2.78
ELK1	2002	ELK1, member of ETS oncogene family	54.72974	0.0007	Fold change	−2.68
PATZ1	23598	POZ (BTB) and AT hook containing zinc finger 1	52.47856	0.0425	Fold change	−2.61
POU5F1	5460	POU class 5 homeobox 1	51.38324	0.0209	Fold change	−2.57
MYC	4609	v-myc myelocytomatosis viral oncogene homologue (avian)	47.85101	0.014	Fold change	2.44
TNFSF14	8740	tumour necrosis factor (ligand) superfamily, member 14	44.25523	0.0363	Fold change	2.32
RORA	6095	RAR-related orphan receptor A	40.17839	0.0104	Fold change	2.19
CABIN1	23523	calcineurin binding protein 1	39.78421	0.0449	Fold change	−2.17
CACTIN	58509	cactin, spliceosome C complex subunit	36.45856	0.0138	Fold change	−2.06
HNF1B	6928	HNF1 homeobox B	34.73115	0.0304	Fold change	−2
TP53	7157	tumour protein p53	34.73115	0.0107	Fold change	−2
MEIS2	4212	Meis homeobox 2	33.6295	0.0219	Fold change	1.96
STAT3	6774	signal transducer and activator of transcription 3 (acute-phase response factor)	32.22958	0.0482	Fold change	−1.92
IKZF2	22807	IKAROS family zinc finger 2 (Helios)	29.0136	0.0436	Fold change	−1.8
TCF7	6932	transcription factor 7 (T-cell specific, HMG-box)	28.50952	0.0391	Fold change	−1.78
EMC10	284361	ER membrane protein complex subunit 10	25.6162	0.0438	Fold change	−1.66
PRMT5	10419	protein arginine methyltransferase 5	25.41787	0.0348	Fold change	−1.65
SETD8	387893	SET domain containing (lysine methyltransferase) 8	25.41787	0.0342	Fold change	−1.65
GFI1	2672	growth factor independent 1 transcription repressor	24.67162	0.0108	Fold change	1.62
DLX2	1746	distal-less homeobox 2	24.26336	0.0361	Fold change	1.6
NFYA	4800	nuclear transcription factor Y, alpha	23.97633	0.0276	Fold change	1.59
NFE2L2	4780	nuclear factor (erythroid-derived 2)-like 2	23.97633	0.0154	Fold change	1.59
NFIC	4782	nuclear factor I/C (CCAAT-binding transcription factor)	23.25614	0.0293	Fold change	−1.55
ESR1	2099	oestrogen receptor 1	23.07699	0.0318	Fold change	−1.54
RXRA	6256	retinoid x receptor, alpha	22.53369	0.0055	Fold change	−1.51

Continued

Supplementary Table SIV *Continued*

Biosets versus Genes record downloaded from Correlation Engine (2017/05/19 04:26:14)
Bioset 1: Endometrial epithelium – sorted side population cells _vs_ unsorted epithelial cells (Study: Side population cells of the human endometrial epithelium and stroma)

https://liverpool.ussc.informatics.illumina.com/c/search/adv.nb?ids=241789

Gene	EntrezGene ID	Gene description	Score	*P*-value	Activity type	Activity value
STAT5A	6776	signal transducer and activator of transcription 5A	21.8965	0.004	Fold change	−1.47
TBL1X	6907	transducin (beta)-like 1X-linked	21.58783	0.0227	Fold change	−1.45
MITF	4286	microphthalmia-associated transcription factor	20.95751	0.0437	Fold change	−1.4
DNAJC2	27000	DnaJ (Hsp40) homologue, subfamily C, member 2	20.58189	0.0187	Fold change	1.37
KLF11	8462	Kruppel-like factor 11	20.58189	0.0002	Fold change	1.37
GTF2A1	2957	general transcription factor IIA, 1, 19/37 kDa	20.38104	0.0186	Fold change	−1.35
SGPL1	8879	sphingosine-1-phosphate lyase 1	20.27312	0.0496	Fold change	−1.34
TBX5	6910	T-box 5	19.36176	0.0137	Fold change	1.22
POU3F1	5453	POU class 3 homeobox 1	19.36176	0.0301	Fold change	1.22
ATP2B2	491	ATPase, Ca++ transporting, plasma membrane 2	19.31292	0.0261	Fold change	−1.21
